# Blind Device Detection via Extended Sparsity Estimation-OMP in Grant-Free NOMA-IoT

**DOI:** 10.3390/s26113560

**Published:** 2026-06-03

**Authors:** Nur Andini, Andriyan Bayu Suksmono, Joko Suryana, Koredianto Usman

**Affiliations:** 1 School of Electrical Engineering and Informatics, Bandung Institute of Technology (ITB), Bandung 40132, West Java, Indonesia; 2 Telecommunication Engineering Study Program, School of Electrical Engineering, Telkom University, Main Campus (Bandung Campus), Jl. Telekomunikasi No. 1, Bandung 40257, West Java, Indonesia

**Keywords:** device detection, extended sparsity estimation-orthogonal matching pursuit (ESE-OMP), grant-free non-orthogonal multiple access (NOMA), internet of things (IoT), multiple measurement vector (MMV), signal reconstruction, single measurement vector (SMV)

## Abstract

Grant-free non-orthogonal multiple access (NOMA) enables communication without a scheduling process. Base station (BS) must detect active users without knowing their number, a challenge that also occurs in grant-free NOMA–Internet of Things (IoT). Device detection in grant-free NOMA-IoT can be considered as signal reconstruction in compressive sensing (CS). To address this limitation, we propose extended sparsity estimation- orthogonal matching pursuit (ESE-OMP) to detect active devices in single measurement vector (SMV) and multiple measurement vector (MMV) problems for grant-free NOMA-IoT systems, a reconstruction method in CS that operates without prior knowledge of the sparsity level, which corresponds to the number of active devices. The algorithm iteratively detects active devices by monitoring the absolute difference in $l_{1}$-norm of successive residuals, terminating when the change falls below a predefined threshold ε. ESE-OMP is evaluated under various grant-free NOMA-IoT systems, irregular low-density spreading-orthogonal frequency division multiplexing (LDS-OFDM), regular LDS-OFDM, and pattern division multiple access (PDMA) systems. When the signal-to-noise ratio (SNR) is 10 dB for the SMV problem with static active device composition, the regular LDS-OFDM system achieves a bit error rate (BER) of $2.95\times 10^{-4}$, while irregular LDS-OFDM and PDMA systems achieve BERs of $3.78\times 10^{-3}$ and $1.79\times 10^{-2}$, respectively. The smaller the number of active devices, the better the performance of ESE-OMP.

## 1. Introduction

Nowadays, wireless communications have evolved to fifth-generation (5G). There are three communication types or use cases in 5G, such as enhanced mobile broadband (eMBB), which is the deployment of new radio (NR), massive machine type communications (mMTC) with massive users or devices, and ultra reliable and low latency communications (URLLC) with low latency [1,2]. eMBB, which involves human-type communications, is generally carried out in downlink direction [3]. Compared to eMBB, mMTC involves machine-type communications with small packet sizes and sporadic transmissions. Communication in mMTC is mostly carried out in uplink direction. Besides that, mMTC is concerned with autonomous communications and is also known as massive internet of things (IoT). The last communication type is URLLC, which is concerned with data reliability and low latency. URLLC is also known as critical IoT. Wireless communications also will be evolved to sixth-generation (6G), and massive IoT is one of its features. This indicates that mMTC will be developed and become the major feature in 6G. Compared to earlier wireless communications, IoT in 5G and 6G can provide machine-type communications. This IoT consists of massive and critical IoT. In mMTC, or massive IoT, latency as the impact of signaling overhead becomes a significant challenge because of the large number of users. This degrades overall system performance and becomes the main focus in this research.

To meet the massive connectivity demands in 5G, a key multiple access technique is non-orthogonal multiple access (NOMA). NOMA has great potential in massive connectivity [4]. With the principle of non-orthogonality, NOMA can serve the large number of users using shared frequency or time compared with orthogonal multiple access (OMA). In other words, NOMA enhances spectral efficiency with a large number of users [5]. NOMA, including power-domain NOMA (PD-NOMA) and code-domain NOMA, is also one of the possible candidates of next generation multiple access (NGMA) because of its principle of non-orthogonality [6]. It indicates that NOMA, as a candidate of NGMA, can also be implemented in 6G. Because of their principle in non-orthogonality, NOMA and NGMA can serve the large number of users. In NOMA, there is a grant-free scheme that allows communication without a scheduling process between users and base station (BS). This scheme can be implemented in the system with large number of users or devices and short-packet transmission, which are considered for NGMA design. A grant-free scheme can improve connectivity, especially in systems with a large number of users or devices, because there is no scheduling process that results in signaling overhead and latency. On the other hand, communication with the scheduling process can be considered as grant-based communication. Implementing a grant-free scheme, rather than a grant-based scheme, can mitigate signaling overhead and reduce latency. In this way, the connectivity between grant-free users or devices and BS consists of uplink resource configuration and data transmission. Hence, grant-free NOMA can serve a large number of users and mitigate signaling overhead.

In mMTC, or massive IoT, users can be considered as machines or devices. At one time, in mMTC, or massive IoT, the number of devices communicating with BS did not exceed 50% of all devices. Therefore, there are sparse devices communicating with BS in this condition. Communication with sparse devices and without a scheduling process can be considered as a compressive sensing (CS) problem. It means that the device detection process can be solved using signal reconstruction-based CS to get the sparse signal. Multicarrier NOMA, such as low-density spreading (LDS), pattern division multiple access (PDMA), and sparse code multiple access (SCMA), has been proposed for 5G multiple access because of its tradeoff in system performance and complexity [7]. LDS and SCMA can be considered as code-domain NOMA [8]. LDS-orthogonal frequency division multiplexing (LDS-OFDM), by another name low density signature-orthogonal frequency division multiplexing, uses low-density signatures to spread symbols in frequency domain [9] and is one of the code-domain NOMA [6]. In LDS-OFDM, the symbols of users are spread in some subcarriers, and the total number of subcarriers is less than the total number of users. This condition is similar to the compression process in CS when the number of measurement vectors is less than the number of sparse vectors.

CS, which is capable of reconstructing a signal with a small number of samples, can be implemented to detect active users without a scheduling process. In mMTC or massive IoT with a grant-free scheme, CS with the principle of signal reconstruction can be implemented as device detection. Numerous reconstruction methods have been developed in CS, including convex optimization, greedy algorithms, thresholding algorithms, combinatorial algorithms, and non-convex minimization [10,11]. Convex optimization and greedy algorithms can be considered as active user detection methods because these types are robust against noise [11]. In grant-free NOMA systems, particularly grant-free NOMA-IoT, the BS detects devices without knowing the number of these devices communicating. Furthermore, BS also has to detect devices rapidly so that the devices can communicate with BS. In CS, sparsity level represents the number of devices communicating with BS. Convex optimization can be implemented to detect devices without knowledge of the sparsity level or number of devices communicating with BS, but this method is difficult to implement in grant-free NOMA-IoT with a large number of devices. This is because convex optimization detects devices based on $l_{1}$-norm minimization, namely by determining solutions with minimum $l_{1}$-norm. Compared to convex optimization, greedy algorithms determine solutions in each iteration and estimate the signal of devices by finding the maximum correlation between sensing matrix columns and residual or measurement vectors. The complexity of convex optimization is approximately $O(M^{2}N^{3})$, whereas the complexity of greedy algorithms such as matching pursuit (MP) and orthogonal matching pursuit (OMP) is O(MNK) with *M* and *N* being the size of the sensing matrix and *K* being the sparsity level [11]. Therefore, greedy algorithms are more computationally efficient than convex optimization and can be considered as a device detection method. One of the greedy algorithms that can be implemented to detect devices is OMP. OMP has better performance than MP as active user detection [12].

**Figure 1:**
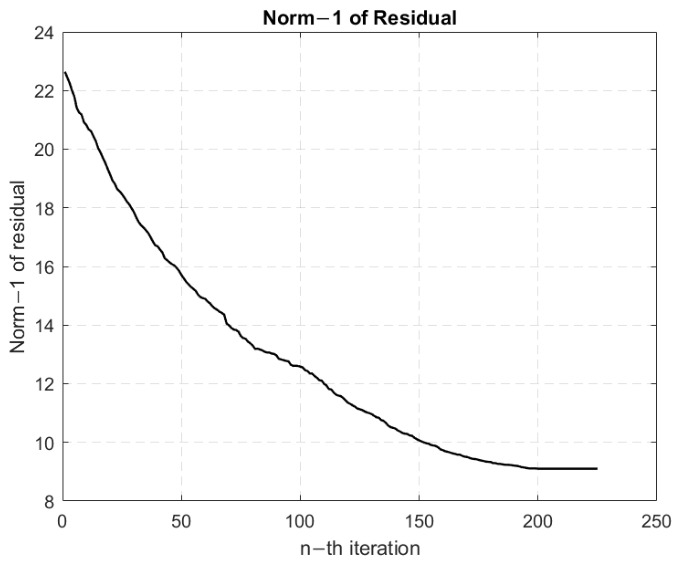
Residual $l_{1}$-norm value of the signal detected with OMP in irregular LDS-OFDM system under Rayleigh fading channel and AWGN effects when SNR of 0 dB. The initial value of residual $l_{1}$-norm is the received measurement vector with time slot-based detection and the sparsity level is 200.

**Figure 2:**
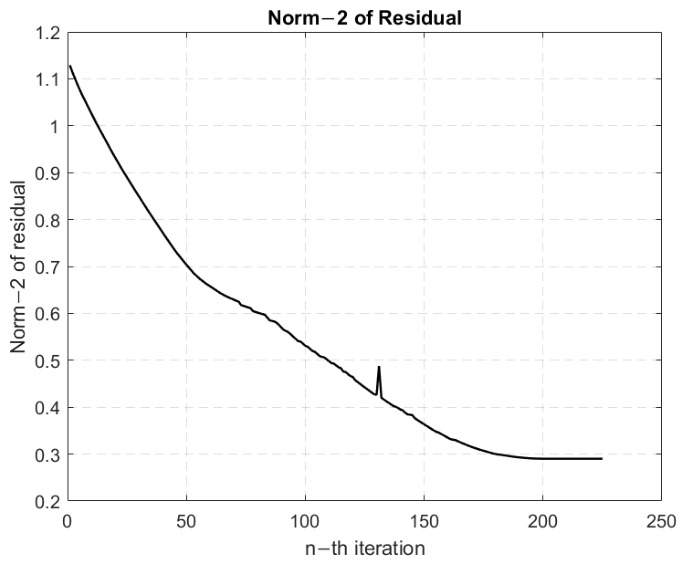
Residual $l_{2}$-norm value of the signal detected with OMP in irregular LDS-OFDM system under Rayleigh fading channel and AWGN effects when SNR of 6 dB. The initial value of residual $l_{2}$-norm is the received measurement vector with time slot-based detection and the sparsity level is 200.

In grant-free NOMA system, communication between active users and BS is established without a scheduling process, and BS does not know how many active users are engaged in communication. Furthermore, the residual norm of OMP, including the $l_{1}$-norm and $l_{2}$-norm, may fluctuate as iterations increase. Figure 1 and Figure 2 show the residual $l_{1}$-norm and $l_{2}$-norm of OMP for signal reconstruction with a sparsity level of 200, respectively. These residual values are affected by communication channel with Rayleigh distribution and additive white Gaussian noise (AWGN) with SNRs of 0 dB for residual $l_{1}$-norm and 6 dB for residual $l_{2}$-norm. After 200 iterations related to sparsity level, the absolute difference of successive residuals is very small. In Figure 2, the residual $l_{2}$-norm fluctuates. In this case, the comparison of current and previous residual $l_{2}$-norm cannot be used to estimate the number of active users. Furthermore, the range of residual $l_{1}$-norm can change, so the residual $l_{1}$-norm threshold can not be used to estimate the number of active users. Therefore, the absolute difference in $l_{1}$-norm of successive residuals can be implemented to determine the OMP iteration for estimating the number of active users. Moreover, the research about active user detection based on CS without prior sparsity level knowledge or number of active users needs to be investigated. This opens the opportunity for developing device detection using CS-based reconstruction methods without prior sparsity level knowledge.

In this paper, we propose the extended sparsity estimation-OMP (ESE-OMP) to estimate the number of devices communicating with BS in grant-free NOMA-IoT. This algorithm also detects devices that are represented by data detection. Next, we call the devices communicating with BS as active devices. In this condition, communication between devices and BS is established without a scheduling process, and BS does not know the number of active devices. The estimation process of the number of active devices is based on iteration of the proposed algorithm. The iteration depends on an absolute value of subtraction of the previous residual $l_{1}$-norm with the current residual $l_{1}$-norm; we call this value ε. We use the absolute value because the residual $l_{1}$-norm may fluctuate as iterations increase, as shown in Figure 1. In this condition, we cannot assume that the residual $l_{1}$-norm decreases as iterations increase. The iteration will be terminated if ε is very small. In initialization process, the initial sparsity level is 0 because in the initial condition, we assume there is no active device communicating with the BS. We apply LDS-OFDM and PDMA as multiple access schemes.

In this research, we evaluate ESE-OMP to detect devices based on time slot and frame. Device detection in a time slot is modeled by single measurement vector (SMV)-based signal reconstruction. In this case, the composition of the active device can be constant or vary. In addition to that, the total number of active devices can also be constant or vary. On the other hand, device detection in frame is modeled by multiple measurement vector (MMV)-based signal reconstruction. In this case, the total number of active devices is constant, but the density of their data varies.

The main contribution of this paper is the estimation of the number of active devices using ESE-OMP. This process is the same as the estimation process of support number in OMP. In general, the amount of support in OMP is known. In this paper, the amount of support that represents the number of active devices is unknown to the BS. The algorithm is based on OMP and is extended to ESE-OMP. The detailed contributions are

1. We propose ESE-OMP as an extension of OMP to estimate the number of support that represents the number of active devices. The estimation process is based on the absolute value of the subtraction of the previous residual $l_{1}$-norm with the current residual $l_{1}$-norm because the residual $l_{1}$-norm may fluctuate as iterations increase. Compared to OMP, which needs to know the prior sparsity level, ESE-OMP does not need to know the prior sparsity level.
2. We model the grant-free NOMA-IoT using LDS-OFDM with irregular and regular schemes as well as PDMA. The similarity between these schemes is the number of shared subcarriers used by some active devices when communicating with the BS. In contrast, the difference is the total number of subcarriers used by all devices.
3. We propose ESE-OMP for the SMV and MMV problems. The SMV problem represents the device detection in a time slot, whereas the MMV problem represents the device detection in a frame.

This paper is organized as follows. Related works are explained in Section 2, and the system model of grant-free NOMA-IoT is described in Section 3. SMV and MMV problems in grant-free NOMA-IoT are described in Section 4. In Section 5, the proposed device detection method based on ESE-OMP is explained. Then, results and analysis as well as the conclusion are explained in Section 6 and Section 7, respectively.

## 2. Related Works

Some studies have investigated the implementation of CS to detect active users in grant-free NOMA systems. Most of these studies have implemented greedy algorithms, and one of these algorithms is OMP. OMP-based CS has been proposed as active user detection with knowledge of the sparsity level [13,14]. The OMP iteration is determined by comparing the current residual $l_{2}$-norm and the previous residual $l_{2}$-norm. This iteration takes place if the current residual $l_{2}$-norm is less than the previous residual $l_{2}$-norm. In this research, the sparsity level is used to determine the number of support values that represent the signals transmitted by active users. In [15], OMP-based CS has also been implemented for user activity and data detection. Moreover, group OMP (GOMP), a variant of OMP, has been implemented as active user detection with knowledge of the sparsity level or the residual energy threshold to terminate the iteration [16].

Sparsity level is also used to terminate the iteration in the algorithms based on OMP [17], modified OMP (MOMP) [18], and amended OMP-union-amended subspace pursuit (AMDOMP-union-AMDSP) [19] to find maximum correlation between sensing matrix columns and residual. In [18,19], comparison of the current residual $l_{2}$-norm and the previous residual $l_{2}$-norm is also used to terminate iteration to find maximum correlation between sensing matrix columns and residual. In [12], OMP is implemented to detect active users with shared subcarriers and knowledge of the number of active users. In this case, BS knows how many active users communicate with. Other research determines sparsity level using cross-validation residuals [20]. The initial value of the sparsity level is 1, and the determination of the sparsity level is terminated based on a comparison of the current residual $l_{2}$-norm and the previous residual $l_{2}$-norm. In [21], the determination of sparsity level is based on the comparison of current residual power with previous residual power or with noise power, where residual power is related to residual $l_{2}$-norm. In [22], CS is implemented in the PDMA system to detect active users. In this research, OMP is compared with compressive sampling matching pursuit (CoSaMP). The performance of OMP is better than the performance of CoSaMP in terms of signal-to-noise ratio (SNR) and bit error rate (BER).

In some studies, MMV has been employed in multiuser detection. MMV-CS is utilized to reduce memory and improve detection speed [23]. In this research, simultaneous OMP (SOMP) is used as multiuser detection, and the signal requires the same support location. MMV is also utilized with OMP in multiuser detection for 5G [15]. In this case, the average correlation between the sensing matrix and residual is used to detect user activity. In [24], MMV is transformed to exploit signal sparsity in joint channel estimation and multiuser detection. The different signals in continuous time slots are detected using the MMV model. In the MIMO-NOMA system, MMV is used to utilize spatial correlation [25].

In some related works, the sparsity level that represents the number of active users is needed as prior knowledge to detect active users who communicate with the BS. This sparsity level is used to terminate the iteration of the algorithm or determine the support values. In reality, BS does not know the number of active users in a grant-free NOMA system. Therefore, research about user detection without prior knowledge of the sparsity level needs to be conducted. Some research implements OMP without prior knowledge of the sparsity level, using comparison of the current residual $l_{2}$-norm and previous residual $l_{2}$-norm; however, the residual $l_{2}$-norm can fluctuate. Therefore, comparison of residual $l_{2}$-norm can not be used to terminate the iteration of OMP without prior knowledge of the sparsity level.

## 3. System Model of Grant-Free NOMA-IoT

**Figure 3:**
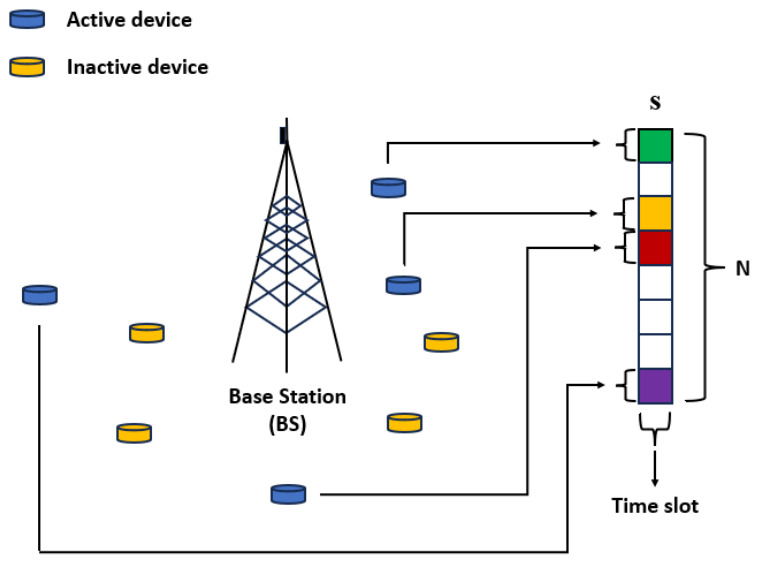
Illustration of BS and device communication in one time slot as the SMV problem. The system consists of one BS and 8 devices with 4 active devices.

We model grant-free NOMA-IoT, which employs one BS, *N* devices, and *M* subcarriers. Therefore, the overloading factor of this system is $\frac{N}{M}$. Devices communicate with the BS without a scheduling process. Generally, there are *K* active devices that share many subcarriers to communicate. The illustration of communication between BS and devices is shown in Figure 3. In this illustration, the total number of devices, *N*, is eight, with the total number of active devices, *K*, being four. In one time slot, there are four active devices that communicate with the BS. The signal of all devices is represented by N×1 vector, **s**. In this scenario, there are four active devices and four inactive devices, so that the sparsity level of **s** is 50%.

In this research, we implement LDS-OFDM and PDMA to model the grant-free NOMA-IoT. The difference between the system with LDS-OFDM and PDMA is the spreading matrix usage. The system with LDS-OFDM uses an LDS spreading matrix, whereas the system with PDMA uses a PDMA matrix. In particular, there is one device that uses all subcarriers to communicate with the BS in a system with PDMA. Next, we call the system with LDS-OFDM the LDS-OFDM system and the system with PDMA the PDMA system.

### 3.1. LDS-OFDM

**Figure 4:**
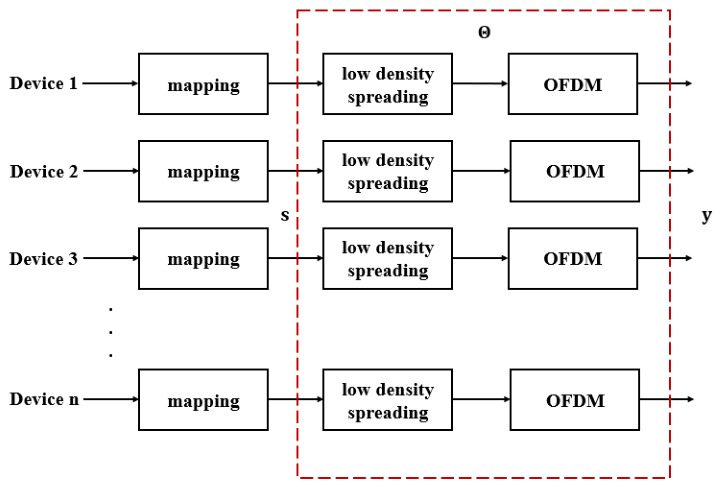
Block diagram of LDS-OFDM transmitter in devices. Low-density spreading and OFDM blocks are considered as compression processes in CS with sensing matrix Θ.

Figure 4 shows the system model of the device as an LDS-OFDM transmitter. Signals of active devices are processed in the mapping block using phase shift keying (PSK), whereas the signals of inactive devices are set to 0. The combined signal from active devices and inactive devices is denoted by **s**. In the LDS-OFDM transmitter, the modulated signal is processed in a low-density spreading block to map active devices to one or two subcarriers. This process is called the spreading process. The illustration of these mapping matrices is shown in $L_{Irregular}$ and $L_{Regular}$ as low-density spreading matrices. In this illustration, there are 8 devices, 6 subcarriers, and 4 active devices that communicate with the BS. Each column of these matrices represents the spreading code used by each device. Additionally, each column represents the subcarriers used by each active device. On the other hand, each row of these matrices represents each subcarrier with the contained devices.

(1) $$L_{Irregular}=\left[\begin{array}{cccccccc} 1 & 0 & 0 & 0 & 0 & 0 & 0 & 0\\ 0 & 0 & 1 & 0 & 0 & 0 & 1 & 0\\ 1 & 0 & 0 & 0 & 0 & 0 & 0 & 1\\ 0 & 0 & 0 & 0 & 0 & 0 & 0 & 0\\ 0 & 0 & 1 & 0 & 0 & 0 & 0 & 0\\ 0 & 0 & 0 & 0 & 0 & 0 & 0 & 0 \end{array}\right]$$

(2) $$L_{Regular}=\left[\begin{array}{cccccccc} 0 & 0 & 0 & 0 & 0 & 1 & 0 & 0\\ 0 & 0 & 0 & 0 & 0 & 0 & 1 & 0\\ 1 & 0 & 0 & 0 & 0 & 0 & 0 & 0\\ 1 & 0 & 0 & 0 & 1 & 0 & 0 & 0\\ 0 & 0 & 0 & 0 & 1 & 0 & 0 & 0\\ 0 & 0 & 0 & 0 & 0 & 1 & 1 & 0 \end{array}\right]$$

**Figure 5:**
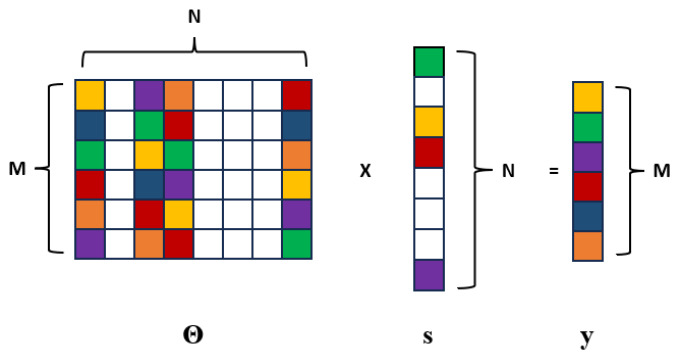
Illustration of matrix multiplication on grant-free NOMA communication in one time slot as an SMV problem. There are 6 subcarriers and 8 devices with 4 active devices.

After the spreading process, the signal is processed in an OFDM block that contains an inverse Fast Fourier Transform (IFFT). The output of this process is a signal sent by devices; this signal is denoted by **y** as shown in Figure 4. The process in the LDS-OFDM transmitter can be considered as a compression process in CS. In CS principle, modulated signal **s** is denoted as a sparse vector, and signal sent by devices **y** is denoted as a measurement vector. Measurement or sensing matrix Θ is the integration of the low-density spreading matrix and the IFFT matrix in LDS-OFDM. In general,

(3) y=Θs,

where $y={\left[y_{1},y_{2},y_{3},\ldots ,y_{M}\right]}^{T}$, $s={\left[s_{1},s_{2},s_{3},\ldots ,s_{N}\right]}^{T}$, and Θ is an M×N measurement or sensing matrix. **s** is a modulated symbol with values of 1 and −1, Θ and **y** are complex values. In more detail, (3) can be written as   

(4) $$y_{m}=\sum_{m=1}^{M}\sum_{n=1}^{N}\theta_{m,n}s_{n},$$

where $y_{m}$ is an element of y that denotes the signal of each subcarrier, $s_{n}$ is an element of s that denotes the signal of each device, and $\theta_{m,n}$ is an element of Θ. Θ contains active devices and a subcarrier mapping matrix. The illustration of matrix multiplication (3) is shown in Figure 5. In this illustration, there are eight devices with four active devices that communicate with BS, and the signal of these devices is represented by the N×1 vector **s**. The M×N sensing matrix is represented by Θ, and the result is an M×1 measurement vector, **y**.

In addition, the process in the device as transmitter based on Figure 4 is described as follows.

1. Active devices generate their data as either 0 or 1.
2. The active device’s signal is processed in the modulation block using binary phase shift keying (BPSK). This is because the communication is carried out with small packet sizes and sporadic transmissions. Therefore, the modulated symbols are 1 and −1 based on the BPSK constellation diagram.
3. Modulated symbol of active devices and signal 0 of inactive devices form **s**.
4. **s** is processed in low density spreading by multiplying with M×N low density spreading **L**. In LDS-OFDM case, **L** can be $L_{Irregular}$ or $L_{Regular}$. Thus,
  (5) x=Ls.
  In more detail, (5) can be written as
  (6) $$x_{m}=\sum_{m=1}^{M}\sum_{n=1}^{N}l_{m,n}s_{n},$$
  where $x_{m}$ is an element of x, $l_{m,n}$ is an element of L, and $s_{n}$ is an element of s, as shown in (3).
5. **x** is processed in the OFDM block by multiplying with M×M inverse of the FFT matrix $W_{M}$. Thus,
  (7) $$y=W_{M}^{-1}x.$$
  In another way, (7) can be written as
  (8) $$y_{m}=\frac{1}{M}\sum_{m=0}^{M-1}x_{m}e^{j2\pi \frac{m}{M}}.$$
  Therefore,
  (9) $$\Theta =W_{M}^{-1}L.$$

LDS matrix denotes the subcarrier utilization by devices. Active devices communicate with the BS using a subcarrier. There are two scenarios of subcarrier mapping during the communication process in the LDS-OFDM system. The difference between these scenarios is the total number of subcarriers used by each active device. Thus, the structure of the LDS matrix **L** and elements of the sensing matrix Θ are different.

**Figure 6:**
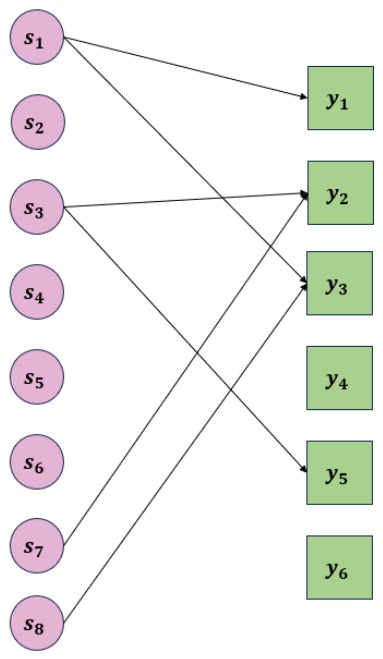
Illustration of device and subcarrier mapping in irregular LDS-OFDM system. There are 6 subcarriers and 8 devices with 4 active devices.

**Figure 7:**
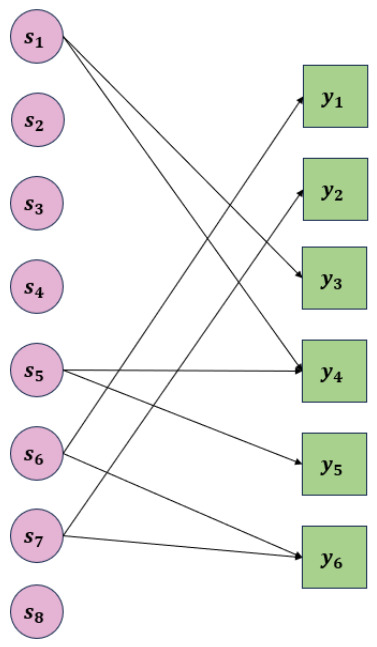
Illustration of device and subcarrier mapping in regular LDS-OFDM system. There are 6 subcarriers and 8 devices with 4 active devices.

1. *K* active devices are mapped on the $\frac{K}{2}$ shared subcarriers. Because all active devices are mapped on the same $\frac{K}{2}$ subcarriers, there are 2 active devices that are mapped on each shared subcarrier. Therefore, $\left(K-\frac{K}{2}\right)$ active devices have to be mapped on the other $\left(K-\frac{K}{2}\right)$ subcarriers. Thus, all active devices are mapped on different subcarriers, and each active device has a unique spreading code. Next, we mention it as an irregular LDS-OFDM system. The illustration of this scenario is described using the matrix $L_{Irregular}$. The matrix represents the LDS matrix with 8 devices, 4 active devices, and 6 subcarriers. Matrix of irregular LDS-OFDM system is illustrated in (1). In this system, there are 2 shared subcarriers that are used by 2 active devices. Illustration of the device and subcarrier mapping of the irregular LDS-OFDM system is shown in Figure 6. In Figure 6, the degree of $\frac{K}{2}$ active devices and $\frac{K}{2}$ subcarriers used is 2. On the other hand, the degree of $\left(K-\frac{K}{2}\right)$ active devices and $\left(K-\frac{K}{2}\right)$ subcarriers is 1. Active devices whose degree is 2 spread their information in two subcarriers; one subcarrier contains only their information, and the other subcarrier contains not only their information but also the information of other active devices. In this case, if one subcarrier is affected by the communication channel, there is one other subcarrier that contains the active device information. On the other hand, active devices whose degree is 1 spread their information only in one subcarrier, and this subcarrier not only contains their information but also one more active device’s information. If the subcarrier is affected by the communication channel, there is no more subcarrier that contains this active device’s information.
2. Each *K* active device is mapped to two subcarriers. In this scheme, there are $\frac{K}{2}$ shared subcarriers containing the information from 2 active devices, and there are *K* subcarriers containing information from 1 active device. Thus, all active devices are mapped on different subcarriers, and each active device has a unique spreading code. Next, we mention it as a regular LDS-OFDM system. An illustration of this scheme is described using the matrix $L_{Regular}$ as shown in (2). In Figure 7, the degree of all active devices is 2. The degree of the $\frac{K}{2}$ subcarrier is 2, and the degree of the *K* subcarrier is 1. All active devices spread their information in two subcarriers with a subcarrier of degree 1 and a subcarrier of degree 2. If the one subcarrier is affected by the communication channel, there is one more subcarrier that contains the active device information. In this case, all subcarriers are used to send active devices information.

During the transmission process, communication channels and noise affect the signal sent by all devices. We model the communication channel using the Rayleigh distribution and noise using AWGN. The devices are in a stationary condition with a velocity of 0 km/h. Thus,

(10) z=Hy+v,

where $z={\left[z_{1},z_{2},z_{3},\ldots ,z_{M}\right]}^{T}$ is the received signal from all devices, $v={\left[v_{1},v_{2},v_{3},\ldots ,v_{M}\right]}^{T}$ is an AWGN vector with Gaussian distribution, and **H** is an M×M communication channel matrix with Rayleigh distribution. **H**, **v**, and **z** are complex values. After this signal is received by the BS, active devices will be detected using ESE-OMP. In this case, channel estimation is not implemented, so the detection process is carried out without channel state information (CSI).

### 3.2. PDMA

**Figure 8:**
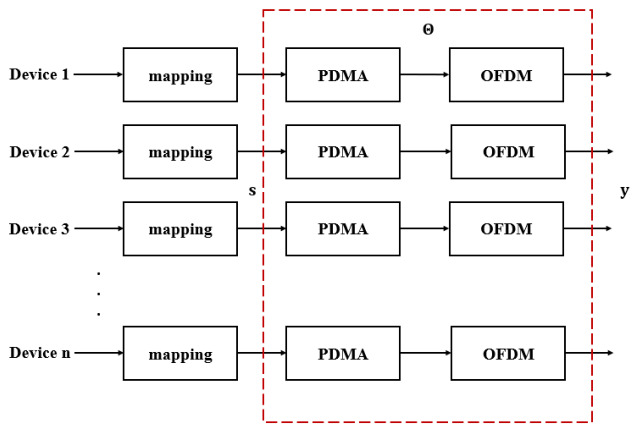
Block diagram of PDMA transmitter in devices. PDMA and OFDM blocks are considered as a compression process in CS with sensing matrix Θ.

**Figure 9:**
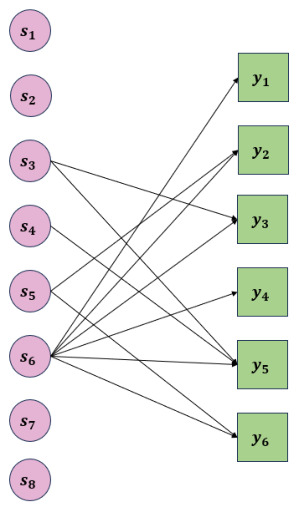
Illustration of device and subcarrier mapping in PDMA system. There are 6 subcarriers and 8 devices, with 4 active devices.

The block diagram of the PDMA transmitter is shown in Figure 8. Same as LDS-ODFM, signals of active devices are processed in the mapping block using BPSK, whereas signals of inactive devices are set to 0, and they form **s**. In the PDMA transmitter, a modulated signal is processed in the PDMA block not only for mapping active devices to one or two subcarriers but also for mapping one active device to all subcarriers. The detailed process is that one active device is mapped on all subcarriers, and other active devices are mapped on one or two subcarriers. The illustration of this mapping matrix is shown as $L_{PDMA}$ in (11). This mapping matrix is L as shown in (5), and its detailed version is shown in (6). There is one column that contains 1 in all rows. The illustration of user and subcarrier mapping is shown in Figure 9. In Figure 9, the degree of K−1 active devices is 2 or 1. Unlike these active devices, 1 active device has the degree of *M*. The signal that has been processed in the PDMA block will be processed in the OFDM block as shown in (7) and (8). The difference between this scenario and the LDS-OFDM system scenario is the measurement or sensing matrix of each scenario.

(11) $$L_{PDMA}=\left[\begin{array}{cccccccc} 0 & 0 & 0 & 0 & 0 & 1 & 0 & 0\\ 0 & 0 & 0 & 0 & 1 & 1 & 0 & 0\\ 0 & 0 & 1 & 0 & 0 & 1 & 0 & 0\\ 0 & 0 & 0 & 0 & 0 & 1 & 0 & 0\\ 0 & 0 & 1 & 1 & 0 & 1 & 0 & 0\\ 0 & 0 & 0 & 0 & 1 & 1 & 0 & 0 \end{array}\right]$$

## 4. Single Measurement Vector (SMV) and Multiple Measurement Vector (MMV) Problems in Grant-Free NOMA-IoT

In this communication, there are some scenarios representing the composition, total number, and data density of active devices. The scenarios with static or dynamic composition and total number of active devices are considered as SMV problems, while the scenario with dynamic data density of active devices is considered as an MMV problem.

### 4.1. SMV Problem

This problem represents communication with detection in a time slot. Figure 3 shows the scenario of communication with detection in a time slot. In the SMV problem, the modulated signal **s** in (3) is the N×1 sparse vector. Multiplication of the M×N sensing matrix Θ and **s** is illustrated in Figure 5. The detection of the estimated signal $\hat{s}$ uses an M×N sensing matrix Θ based on (3). In this problem, there are three scenarios as follows.

1. Static composition of active devices is the scenario in which the total number of active devices, *K*, is constant. In this scenario, the composition of the active device is fixed. Device detection is carried out in a time slot with a fixed composition. The M×N sensing matrix is fixed during data transmission.
2. Dynamic composition of active devices is the scenario in which the total number of active devices is constant, but the composition of active devices varies in each time slot. In other words, it is the transmission of some sequential time slots. The total number of active devices, *K*, is 200 from the total number of devices, *N*, 400. Once the time slot changes, the sparse vector **s** changes. The combination of sparse vector **s** is CKN=400200. The M×N sensing matrix changes during data transmission based on the position of active devices.
3. Dynamic total number of active devices is the scenario in which the total number of active devices varies with the numbers 40, 80, 120, 160, and 200. For instance, at one time, the total number of active devices is 80, but at another time, the total number of active devices is 200. The M×N sensing matrix changes during data transmission based on the total number and position of active devices. The device detection in this scenario can be considered an SMV problem, where detection is carried out in each time slot.

### 4.2. MMV Problem

**Figure 10:**
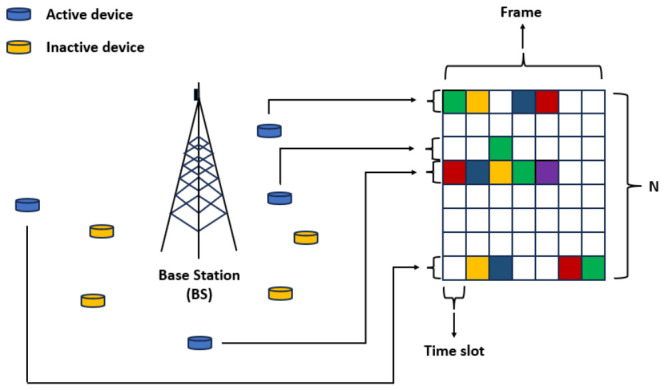
Illustration of BS and devices communication in one frame as the MMV problem. The system consists of one BS and 8 devices with 4 active devices. The frame consists of 7 time slots.

This problem represents the data density of active devices in one frame when they communicate with the BS, as illustrated in Figure 10. In this scenario, the total number and composition of active devices are constant, but the data density of each active device in one frame is different. Each active device does not send its information continuously throughout some time slots or frames. There are (N−K) inactive devices that have not sent data for a long time. The total number of active devices in this scenario is 200 of 400, the total number of devices. The sparse signal **s** in (3) is the N×7 matrix with the total number of time slots in one frame, *G*, being 7. In one frame, because each active device does not always send the data, each row of the sparse signal **s** does not contain data completely. In this case, we evaluate the performance of ESE-OMP when active devices send data in one frame. The sensing matrix generation in the MMV problem is the same as the sensing matrix generation in the SMV problem for all systems: irregular LDS-OFDM, regular LDS-OFDM, and PDMA systems.

**Figure 11:**
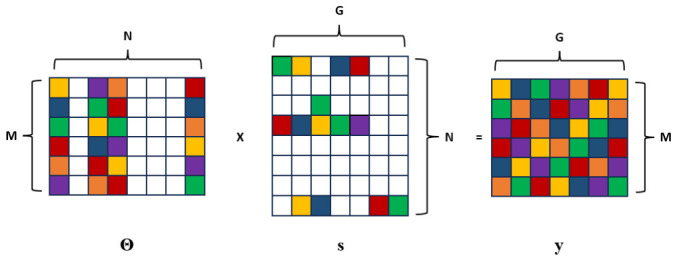
Illustration of matrix multiplication on grant-free NOMA communication in one frame as MMV problem. There are 6 subcarriers and 8 devices with 4 active devices. Frame consists of 7 time slots.

Same as the static composition scenario, the M×N sensing matrix Θ is fixed during the data transmission for one frame. The difference lies in the form and size of the sparse signal **s**, and this difference causes the difference in form and size of measurement **y**. Once a sparse signal is an N×1 vector, the measurement **y** becomes an M×1 vector in the SMV problem. In the MMV problem, the sparse signal is an N×7 matrix, and measurement **y** is also an M×7 matrix. Figure 11 shows the illustration of matrix multiplication in the MMV problem.

## 5. Proposed Device Detection Method Based on ESE-OMP

**Figure 12:**
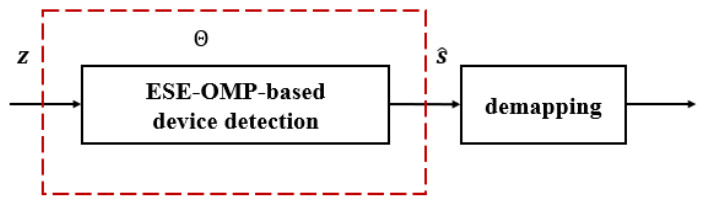
Block diagram of receiver based on reconstruction method in BS. ESE-OMP is implemented to detect active devices with the recontruction signal principle.

The detection process of devices is carried out using the proposed method, ESE-OMP. ESE-OMP is a reconstruction method that is based on OMP. The block diagram of BS as receiver with ESE-OMP is shown in Figure 12. The received signal **z** is processed in the ESE-OMP-based device detection block based on a reconstruction method with an M×N sensing matrix Θ. In this case, the received signal **z** has been affected by a communication channel with a Rayleigh distribution and AWGN. In the proposed method, the received signal is detected without communication channel mitigation before the reconstruction method. In the SMV problem, **z** is an M×1 vector, whereas in the MMV problem, **z** is an M×7 matrix.

OMP is one of the reconstruction algorithms that recovers a *K*-sparse signal using the greedy principle. The *K*-sparse signal with a length of *N* consists of *K* non-zero elements and N−K zero elements as follows.

(12) $$s_{k}\left\{\begin{array}{c} \neq 0,k=1,2,...,K\\ =0,k=K+1,...,N \end{array}\right.$$

**Figure 13:**
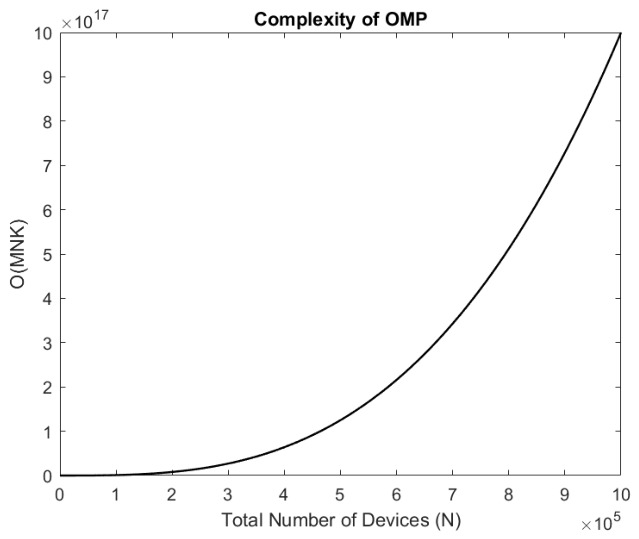
OMP complexity based on Big O notation. The greater the number of devices, the greater the OMP complexity.

In this research, we consider *K*-sparse **s** with *K* non-zero elements that represent the number of active devices. In OMP, non-zero elements form the support $supp\left(x\right)=\left\{i|x_{i}\neq 0\right\}$, so the cardinality of the support is *K*. The complexity of OMP is O(MNK), which means that the complexity increases with the increase of *M*, *N*, or *K*. Once the number of *N* increases, *M* and *K* as functions of *N* also increase. Then, the complexity as the result of O(MNK) increases as shown in Figure 13.

**Figure 14:**
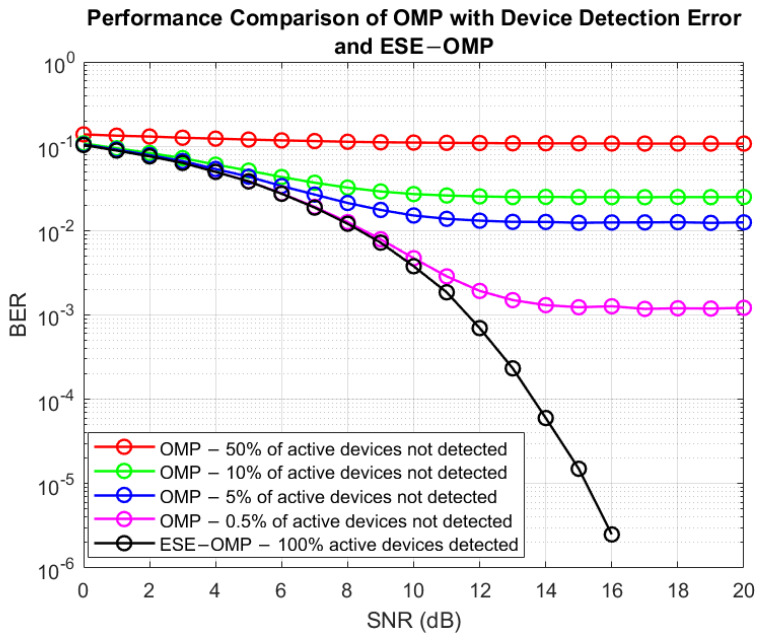
Device detection error with OMP. The error occurs when there is at least 1 active device not detected.

The estimated signal $\hat{s}$ can be well reconstructed if the restricted isometry property (RIP) is achieved. The RIP is expressed as [13]

(13) $$\left(1-\delta_{s}\right){∥s∥}_{2}^{2}\leq {∥\Theta s∥}_{2}^{2}\leq \left(1+\delta_{s}\right){∥s∥}_{2}^{2},$$

where $\delta_{s}$ is the constant with a value of $0\leq \delta_{s}<1$. ESE-OMP has to estimate the number of active devices and detect the data of active devices correctly. If ESE-OMP estimates the number of active devices incorrectly, the data of active devices will also be detected incorrectly, and system performance will decrease. For instance, if there are 200 active devices and ESE-OMP estimates 199 active devices, system performance will decrease. Figure 14 shows system performance with estimation error when ESE-OMP can not estimate the number of active devices in an irregular LDS-OFDM system. If 1 active device or 0.5% of active devices is not detected to communicate with the BS, the data of this active device will not be detected. Therefore, the system performance will result in a high BER. After an SNR of 15 dB, when 10 active devices or 5% of active devices are not detected to communicate with the BS, the achieved BER is 10 times the achieved BER when 1 active device is not detected. This also applies to the condition when 10% and 50% of active devices are not detected to communicate with the BS; the achieved BER will increase according to the increase in the number of active devices that are not detected. The achieved BER of the system with estimation error is very high when compared to the system with correct estimation.

OMP consists of three mandatory processes, such as the determination of maximum correlation between residual and sensing matrix to identify the support, *K*-sparse signal detection using least square (LS), and the residual calculation. The result of the residual value will be used in the next iteration to signal detection process. In detail, a signal is detected by determining the *K* maximum correlation between residual and sensing matrix columns. *K* maximum correlations are determined iteratively, and the value of *K* is known in advance. In this algorithm, the value of *K* has to be known as an initial value because this value is the determining factor of the iteration. Therefore, the iterations will be repeated *K* times. In grant-free NOMA-IoT, the BS does not know the number of active devices, so the sparsity level is unknown. ESE-OMP can be implemented to estimate the number of active devices and detect active device data.

### 5.1. ESE-OMP for SMV Problem

**Figure 15:**
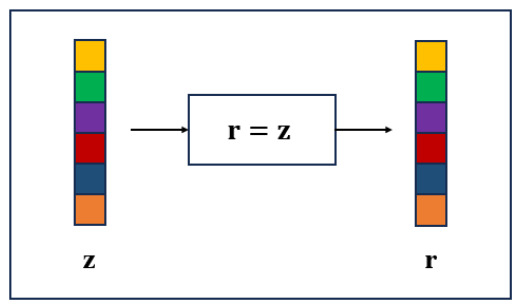
Residual value determination on ESE-OMP for SMV problem. Initial residual value is obtained by measurement vector **z**.

In this research, we propose ESE-OMP-based device detection with the principle of signal reconstruction. Compared to the iteration of OMP that is based on the sparsity level, the iteration of ESE-OMP is based on the absolute value of the subtraction of the previous residual $l_{1}$-norm with the current residual $l_{1}$-norm. In the SMV problem, ESE-OMP-based device detection with the principle of signal reconstruction is shown in Algorithm 1. In the initialization process, the residual value is obtained from z as illustrated in Figure 15.
**Algorithm 1** Extended sparsity estimation-orthogonal matching pursuit (ESE-OMP) for SMV Problem1:Input2:M×N sensing matrix Θ3:M×1 measurement vector z4:⠀5:Initialization6:r=z; ind=⌀; corr=⌀; k=1;7:j=1,2,...,N;8:$d={∥z∥}_{1}$; S=0;9:⠀10:Iteration11:**while** (d>ε) **do**12:   $corr_{k}=\arg\max_{j\setminus ind}\left|\langle r,\Theta_{j}\rangle \right|$13:   ind=ind∪j14:   $\Theta_{p}=\Theta_{ind}$15:   $\hat{s}={\left[\Theta_{p}^{H}\Theta_{p}\right]}^{-1}\Theta_{p}^{H}z$16:   $r_{k}=z-\Theta_{p}\hat{s}$17:   $r_{k-1}=r$18:   $r=r_{k}$19:   S=S+120:   **if** (S=1) **then**21:     $d=\left|{∥z∥}_{1}-{∥r_{k}∥}_{1}\right|$22:   **else**23:     $d=\left|{∥r_{k-1}∥}_{1}-{∥r_{k}∥}_{1}\right|$24:   **end if**25:   k=k+126:**end while**27:⠀28:Output29:Estimated sparsity level $S_{est}=S-1$30:Estimated signal $\hat{s}$ based on sparsity level $S_{est}$

The process of the algorithm is described as follows.

1. (Step 11) In this proposed method, the iteration is based on absolute difference in $l_{1}$-norm of successive residuals or *d*. In initialization process, the value of *d* is the $l_{1}$-norm of z. The value of *d* is bounded by ε, which is a very small value, and ε is set to $10^{-13}$.
2. (Step 12) In every *k*-th iteration, maximum correlation between residual r and column of Θ is calculated with *j* outside ind:
  (14) $$corr_{k}=\arg\max_{j\setminus ind}\left|\langle r,\Theta_{j}\rangle \right|,$$
  where $corr_{k}$ is the correlation value in the *k*-th iteration. The ind value represents the column position of Θ that has maximum correlation with residual r in previous iteration. In the initialization process, ind is an empty matrix.
3. (Step 13) The value of ind is the union of ind in previous iteration and *j*, where the maximum correlation is found.
4. (Step 14) In this step, columns of Θ that have maximum correlation with residual r in every iteration are collected to form a new sensing matrix $\Theta_{p}$.
5. (Step 15) The estimated signal $\hat{s}$ is obtained using LS method:
  (15) $$\hat{s}={\left[\Theta_{p}^{H}\Theta_{p}\right]}^{-1}\Theta_{p}^{H}z.$$
6. (Step 16) Residual r is updated by subtracting z with multiplication of new sensing matrix $\Theta_{p}$ and the estimated signal $\hat{s}$ in each iteration:
  (16) $$r_{k}=z-\Theta_{p}\hat{s}.$$
  The $l_{1}$-norm of residual r tends to decrease as iteration increases. It is because residual is obtained by subtracting z, which is the representation of y with the multiplication of the new sensing matrix $\Theta_{p}$ and the estimated signal $\hat{s}$. The absolute value of the subtraction of the previous residual $l_{1}$-norm with the current residual $l_{1}$-norm will be very small after the sparsity level has been obtained. When SNR is 0 dB, the $l_{1}$-norm of residual r is shown in Figure 1.
7. (Step 19) In this step, sparsity *S*, which is used to conclude the number of active devices, is updated.
8. (Step 20 & 21) In the first iteration, *d* is updated by subtracting the $l_{1}$-norm of z with the $l_{1}$-norm of the current residual r that is obtained in this iteration.
  (17) $$d=\left|{∥z∥}_{1}-{∥r_{k}∥}_{1}\right|.$$
9. (Step 23) In the next iteration, *d* is updated by subtracting the $l_{1}$-norm of the previous residual r that is obtained in previous iteration with the $l_{1}$-norm of the current residual r that is obtained in this iteration.
  (18) $$d=\left|{∥r_{k-1}∥}_{1}-{∥r_{k}∥}_{1}\right|.$$
  After the *d* is not greater than ε, iteration is terminated.

The decision of estimated sparsity level $S_{est}$ is determined by subtracting the sparsity *S* from 1. It is because when the actual sparsity level is obtained, the *d* is not yet very small, or there is a significant difference between the previous and current residual $l_{1}$-norm. This condition causes the iteration to have to be continued once again until *d* is very small, and the iteration is terminated. Once the estimated sparsity level is obtained, the estimated signal $\hat{s}$ can be obtained.

### 5.2. ESE-OMP for MMV Problem

**Figure 16:**
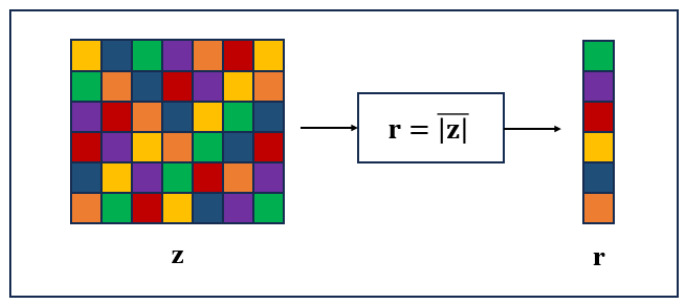
Residual value determination on ESE-OMP for MMV problem. Initial residual value is obtained by the average of absolute value of measurement vector **z**.

In the MMV problem, the input of ESE-OMP as a detection method that is based on signal reconstruction is the M×7 received signal or **z**. In contrast to the SMV problem, in this MMV problem, the finding of maximum correlation is imposed on the average of the absolute value of **z** components. In other words, we calculate the average of the absolute value of **z** components in a frame. Hence, the M×7 received signal or **z** as a matrix becomes an M×1 vector as illustrated in Figure 16. In this case, **r** is obtained by the average of the absolute value of **z** as shown in (19).

(19) $$r_{m}=\frac{1}{G}\sum_{g=1}^{G}\left|z_{m,g}\right|,$$

where $r_{m}$ is element of $r={\left[r_{1},r_{2},r_{3},\ldots ,r_{M}\right]}^{T}$ and $z_{m,g}$ is element of z. Detection process ESE-OMP in MMV problem is shown in Algorithm 2.
**Algorithm 2** Extended sparsity estimation-orthogonal matching pursuit (ESE-OMP) for MMV Problem1:Input2:M×N sensing matrix Θ3:M×7 measurement vector z4:⠀5:Initialization6:$z_{avg}=\bar{\left|z\right|}$; $r=z_{avg}$; ind=⌀; corr=⌀; k=1;7:j=1,2,...,N;8:$d={∥z_{avg}∥}_{1}$; S=0;9:⠀10:Iteration11:**while** (d>ε) **do**12:   $corr_{k}=\arg\max_{j\setminus ind}\left|\langle r,\Theta_{j}\rangle \right|$13:   ind=ind∪j14:   $\Theta_{p}=\Theta_{ind}$15:   ${\hat{s}}_{res}={\left[\Theta_{p}^{H}\Theta_{p}\right]}^{-1}\Theta_{p}^{H}z_{avg}$16:   $\hat{s}={\left[\Theta_{p}^{H}\Theta_{p}\right]}^{-1}\Theta_{p}^{H}z$17:   $r_{k}=z_{avg}-\Theta_{p}{\hat{s}}_{res}$18:   $r_{k-1}=r$19:   $r=r_{k}$20:   S=S+121:   **if** (S=1) **then**22:     $d=\left|{∥z_{avg}∥}_{1}-{∥r_{k}∥}_{1}\right|$23:   **else**24:     $d=\left|{∥r_{k-1}∥}_{1}-{∥r_{k}∥}_{1}\right|$25:   **end if**26:   k=k+127:**end while**28:⠀29:Output30:Estimated sparsity level $S_{est}=S-1$31:Estimated signal $\hat{s}$ based on sparsity level $S_{est}$

The process of the algorithm in the MMV problem is described as follows.

1. (Step 11) Similar to the SMV problem, the iteration is based on the absolute difference in $l_{1}$-norm of successive residuals or *d*. The difference is in the initialization process; the value of *d* is the $l_{1}$-norm of $z_{avg}$. The value of *d* is also bounded by a very small value ε that is set to $10^{-13}$.
2. (Step 12) In initialization process, residual r is set to the average of the absolute value of **z** components. Hence, maximum correlation between residual r and the column of Θ is imposed on the average value. The finding process of maximum correlation is based on (14) calculated with *j* outside ind.
3. (Step 13 & 14) are the same as (Step 13) and (Step 14) in the SMV problem.
4. (Step 15) In this step, the estimated signal ${\hat{s}}_{res}$ is calculated to determine the residual value.
  (20) $${\hat{s}}_{res}={\left[\Theta_{p}^{H}\Theta_{p}\right]}^{-1}\Theta_{p}^{H}z_{avg}.$$
5. (Step 16) The estimated signal $\hat{s}$ is obtained using the LS method that is shown in (15) with z as the M×7 matrix.
6. (Step 17) Residual r is updated by subtracting $z_{avg}$ with multiplication of new sensing matrix $\Theta_{p}$ and the estimated signal ${\hat{s}}_{res}$ in each iteration:
  (21) $$r_{k}=z_{avg}-\Theta_{p}{\hat{s}}_{res}.$$
7. (Step 20) Same as SMV problem, sparsity *S* is updated in this step.
8. (Step 21 & 22) In the first iteration, *d* is updated by subtracting $l_{1}$-norm of $z_{avg}$ with $l_{1}$-norm of current residual r that is obtained in this iteration.
  (22) $$d=\left|{∥z_{avg}∥}_{1}-{∥r_{k}∥}_{1}\right|.$$
9. (Step 24) In the next iteration, *d* is updated using (18). After the *d* is not greater than ε, iteration is terminated.

The decision of estimated sparsity level $S_{est}$ is determined by subtracting the sparsity *S* from 1. This step is the same as the last process in Algorithm 1. This estimated sparsity level can be used to determine the estimated signal $\hat{s}$.

## 6. Results and Analysis

In this research, ESE-OMP is implemented as device detection with the principle of signal reconstruction for SMV and MMV problems. ESE-OMP can estimate the number of active devices communicating with the BS and detect their data. Table 1 shows the simulation parameters. The number of all devices, *N*, is 400, and the number of all subcarriers, *M*, is 300. Therefore, the overloading factor is $\frac{4}{3}$. The number of active devices varies from 40, 80, 120, 160, and 200. Data of active devices is mapped by BPSK, where one symbol consists of one bit of data.

### 6.1. ESE-OMP Performance in SMV Problem

#### 6.1.1. Time Slot-Based Detection with Static Composition of Active Device

**Figure 17:**
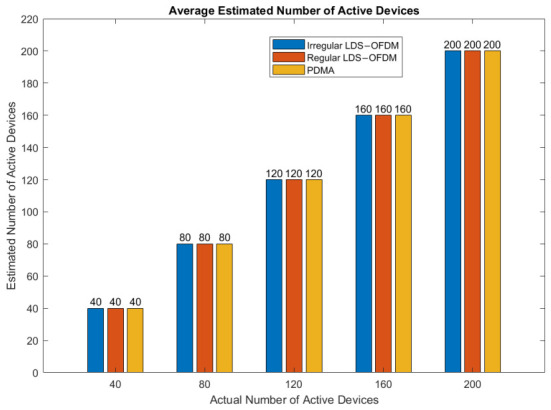
Average estimated number of active devices with ESE-OMP for the SMV problem with time slot-based detection. Variation of the number of active devices evaluated is 40, 80, 120, 160, and 200.

Figure 17 shows that ESE-OMP can estimate the number of active devices correctly when there are 40, 80, 120, 160, and 200 active devices communicating with BS in irregular LDS-OFDM, regular LDS-OFDM, and PDMA systems. These average estimated numbers of active devices are obtained when the SNRs are 0 to 30 dB. In other words, active devices can be detected properly based on the SNRs with ESE-OMP, and the sparsity level can be estimated with an accuracy of 100%, as shown in Table 2. Therefore, in the worst condition, when SNR is 0 dB, or the device power and noise power are the same, ESE-OMP can estimate the whole number of active devices in irregular LDS-OFDM, regular LDS-OFDM, and PDMA systems.

Basically, ESE-OMP performance is the same as OMP performance in terms of SNR and BER, but in ESE-OMP, the sparsity level is unknown. In OMP, the sparsity level has to be known by the BS to detect active devices using the reconstruction method. In grant-free NOMA-IoT, the BS does not know the number of active devices. Hence, there is no prior sparsity knowledge to reconstruct the device data in ESE-OMP. It represents the communication without a scheduling process before the device communicates with the BS. ESE-OMP has to estimate the number of active devices before detecting their data. Therefore, in ESE-OMP, sparsity level estimation and signal reconstruction are carried out simultaneously. The estimation of the sparsity level is represented by the iteration of Algorithm 1. If the absolute value of the subtraction result of the previous residual $l_{1}$-norm with the current residual $l_{1}$-norm, as stated in (18), is almost 0, the iteration is terminated. After the iteration is terminated, the sparsity level is determined by subtracting sparsity *S* from 1. It is because, as long as the sparsity level has not been obtained, the absolute difference between the previous residual $l_{1}$-norm and the current residual $l_{1}$-norm, or *d*, does not approach 0, so the iteration takes place until *d* is very small. With ESE-OMP, BS estimates the number of active devices and detects their data simultaneously, and it is compatible with implementation in grant-free NOMA-IoT. In this condition, detection of active devices includes data detection of these devices. The system performance is compared in terms of SNR and BER.

**Figure 18:**
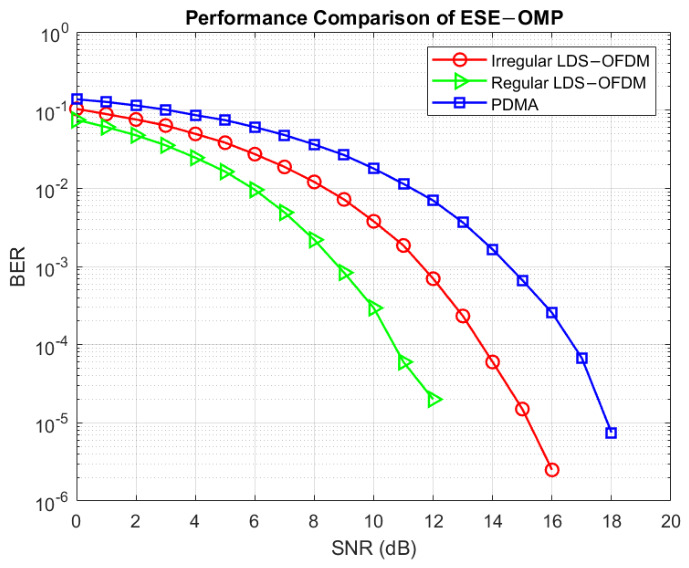
ESE-OMP performance with the number of active devices of 200 for SMV problem with time slot-based detection and static active device composition. The systems evaluated are irregular LDS-OFDM, regular LDS-OFDM, and PDMA systems.

System performance with ESE-OMP as device detection in one time slot, including active device number estimation and data detection, is shown in Figure 18. In Figure 18, ESE-OMP estimates the number of active devices correctly and then detects their data when the number of active devices is 200 for irregular LDS-OFDM, regular LDS-OFDM, and PDMA systems. Generally, the higher the SNR, the lower the achieved BER. The regular LDS-OFDM system has the best performance, whereas the PDMA system has the worst performance because of the number of subcarriers that are used by each active device. In a regular LDS-OFDM system, information of all active devices is spread across two subcarriers. Therefore, all active devices use the same number of subcarriers. In this condition, the symbols of each active device are conceived in two subcarriers. In the irregular LDS-OFDM system, there are $\frac{K}{2}$ active devices that use one subcarrier and $\frac{K}{2}$ active devices that use two subcarriers. Therefore, the signal regeneration in the regular LDS-OFDM system is better than the signal regeneration in the irregular LDS-OFDM system. On the other hand, in the regular LDS-OFDM system, $\frac{K}{2}$ subcarriers contain information from 2 active devices, and *K* other subcarriers contain information from 1 active device. If one subcarrier is decayed by the communication channel, there is still one other subcarrier that contains active device information. This one other subcarrier that is not decayed by the communication channel can be used to recover active device information. The irregular LDS-OFDM system has worse performance than the regular LDS-OFDM system and better performance than the PDMA system. In the irregular LDS-OFDM system, only $\frac{K}{2}$ active devices spread their information to two subcarriers. One of these subcarriers contains only information of 1 active device, whereas the other subcarrier contains not only information of 1 active device but also information of other active device. The other $\frac{K}{2}$ active devices spread their information to one subcarrier, and this subcarrier contains information of 2 active devices. If this subcarrier is decayed by the communication channel, the information of the active device that spread its information only to this subcarrier is also decayed. Therefore, the data detection of this process results in an error. The PDMA system has the worst performance because there is one active device spreading its information to all subcarriers. When SNR is 10 dB, the achieved BER of the regular LDS-OFDM system is $2.95\times 10^{-4}$, the achieved BER of the irregular LDS-OFDM system is $3.78\times 10^{-3}$, and the achieved BER of the PDMA system is $1.79\times 10^{-2}$. The achieved BER of regular LDS-OFDM is approximately 10 times better than the achieved BER of the irregular LDS-OFDM system and 100 times better than the achieved BER of the PDMA system.

**Figure 19:**
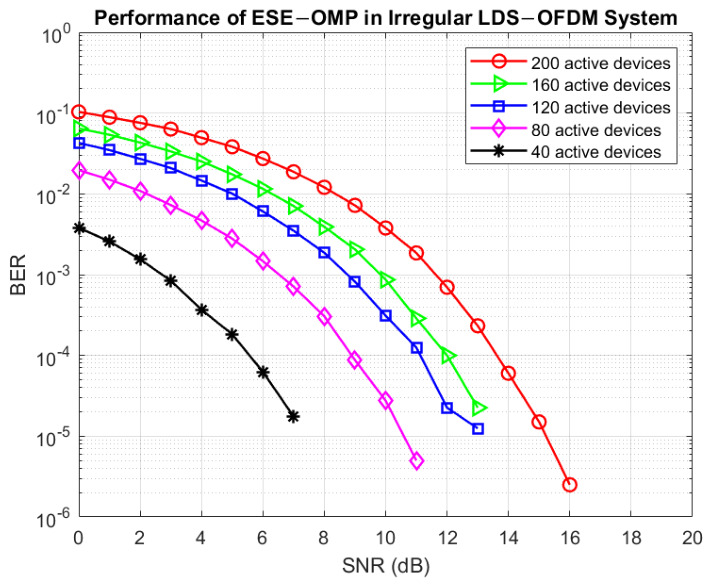
ESE-OMP performance in irregular LDS-OFDM system with varied active devices. Variation of the number of active devices evaluated are 40, 80, 120, 160, and 200.

ESE-OMP can estimate the number of active devices correctly because the absolute difference in the $l_{1}$-norm of successive residuals will be very small after the sparsity level has been obtained, as shown in Figure 1. ESE-OMP performance with varied active devices in an irregular LDS-OFDM system is shown in Figure 19. When the number of active devices increases, the achieved BER also increases in the same SNR. In this case, when the number of active devices increases, the number of shared subcarriers that are used by 2 active devices also increases. For instance, when the number of active devices is 200, the number of shared subcarriers used by 2 active devices is 100, and the number of subcarriers used by only 1 active device is also 100. It means that the total number of subcarriers used in communication is 200 out of 300 subcarriers. In contrast, when the number of active devices is 160, the number of shared subcarriers used by 2 active devices is 80, and the number of subcarriers used by only 1 active device is also 80. It means that the total number of subcarriers used in communication is 160 out of 300 subcarriers. Therefore, the performance of the irregular LDS-OFDM system with 200 active devices and 160 active devices is different. In other words, an irregular LDS-OFDM system with 200 active devices requires a higher SNR than an irregular LDS-OFDM system with 160 active devices to achieve the same BER. In this system, ESE-OMP can estimate the number of active devices correctly when there are 40, 80, 120, 160, and 200 active devices, so the data detection process is conducted on active device data only. Generally, the smaller the number of active devices, the better the performance of ESE-OMP to detect these active devices. Because the number of active devices is represented by the sparsity level, a system with a lower sparsity level needs a lower SNR than a system with a higher sparsity level to achieve a certain BER. It is also because all devices are assumed to transmit their data to the BS, and the BS does not know the number of active devices in grant-free NOMA-IoT. For instance, an irregular LDS-OFDM system with 160 active devices needs a lower SNR than an irregular LDS-OFDM system with 200 active devices to achieve a certain BER. In other words, the achieved BER of a system with 160 active devices is less than the achieved BER of a system with 200 active devices in the same SNR. When a system with 40 active devices needs an SNR of 6 dB, the achieved BER is $6.25\times 10^{-5}$. In the same SNR, systems with 80 and 120 active devices can achieve BERs of $1.47\times 10^{-3}$ and $6.06\times 10^{-3}$, respectively. Systems with 160 and 200 active devices can achieve BERs of $1.16\times 10^{-2}$ and $2.73\times 10^{-2}$, respectively.

**Figure 20:**
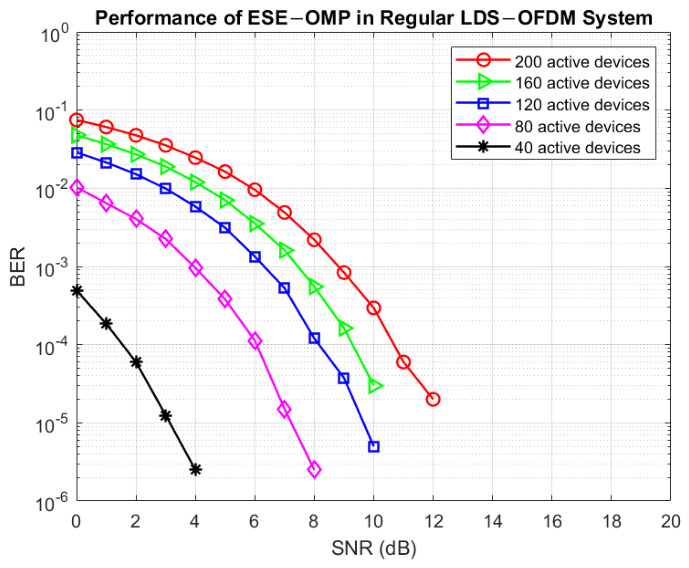
ESE-OMP performance in regular LDS-OFDM system with varied active devices. Variation of the number of active devices evaluated are 40, 80, 120, 160, and 200.

The performance of the regular LDS-OFDM system with varied active devices is shown in Figure 20. In this system, when the number of active devices is 200, the number of shared subcarriers used by 2 active devices is 100, and the number of subcarriers used by only 1 active device is 200. In contrast, when the number of active devices is 160, the number of shared subcarriers used by 2 active devices is 80, and the number of subcarriers used by only 1 active device is 160. The smaller the number of active devices, the better the system performance. In a regular LDS-OFDM system, each active device uses two subcarriers to spread its information. Therefore, the system performance is better than the irregular LDS-OFDM system performance with the same number of active devices. This system is more resistant to the impact of communication channels than the irregular LDS-OFDM system. The achieved BER of the system with 40 active devices is $2.5\times 10^{-6}$ when this system needs an SNR of 4 dB. In contrast to a system with 40 active devices, a system with 80 active devices can achieve a BER of $9.6\times 10^{-4}$ when it needs an SNR of 4 dB. In the same SNR, a system with 120 active devices achieves a BER of $5.83\times 10^{-3}$.

**Figure 21:**
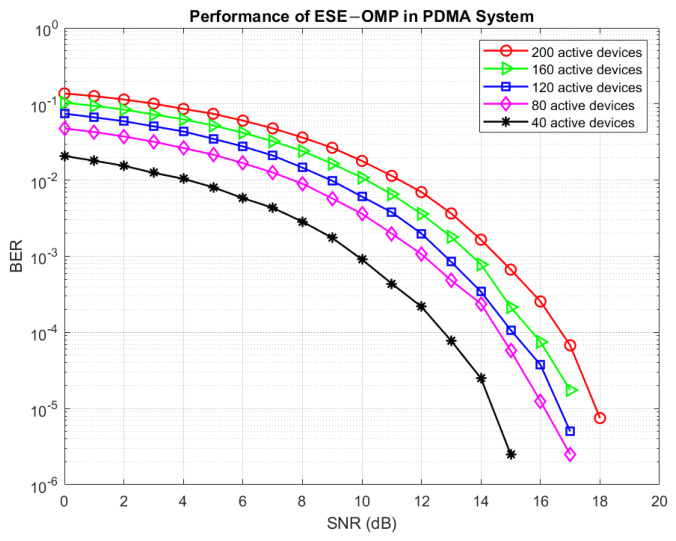
ESE-OMP performance in PDMA system with varied active devices. Variation of the number of active devices evaluated are 40, 80, 120, 160, and 200.

The performance of the PDMA system with ESE-OMP and a varied number of active devices is shown in Figure 21. In this system, there is 1 active device that uses all subcarriers to spread its information. There are three types of active devices in this system: active devices that use one subcarrier, two subcarriers, and all subcarriers. Every subcarrier can be used by a different number of active devices. Each subcarrier contains information from at least 1 active device. In this system, there is one subcarrier that is used by 3 active devices, but in another system, the maximum number of active devices that share one subcarrier is 2. Therefore, the number of active devices sharing one subcarrier in the PDMA system is greater than the number of active devices sharing one subcarrier in irregular LDS-OFDM and regular LDS-OFDM systems. Thus, the performance of the PDMA system with ESE-OMP is the worst. The system performance with 80, 120, and 160 active devices is almost the same.

**Figure 22:**
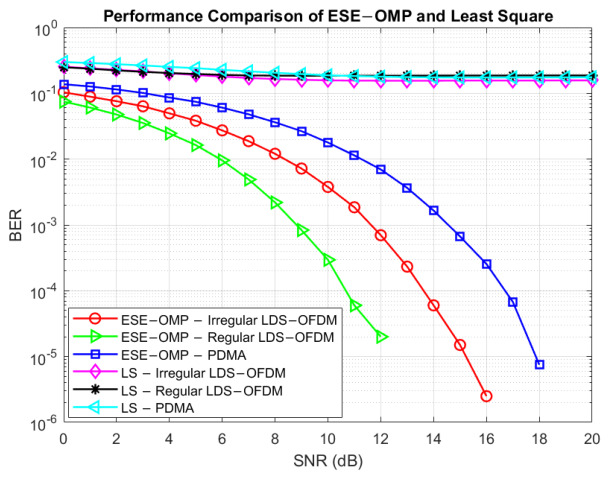
Performance comparison of ESE-OMP and LS for SMV problem with time slot-based detection and static active device composition. The systems evaluated are irregular LDS-OFDM, regular LDS-OFDM, and PDMA systems.

Figure 22 shows the performance comparison of ESE-OMP and LS as device detection in irregular LDS-OFDM, regular LDS-OFDM, and PDMA systems. LS cannot estimate the number of active devices communicating with BS, whereas ESE-OMP can estimate it. Because LS cannot estimate the number of active devices, the data of active devices cannot be detected properly. Compared to LS, in ESE-OMP, there is the determination of maximum correlation between residual and sensing matrix columns, $\left|\langle r,\Theta_{j}\rangle \right|$, so that the number of active devices can be estimated. This process can increase the performance of the detection process because this process forms the new sensing matrix that contains some columns of the sensing matrix with maximum correlation with residual r. In terms of BER and SNR, the achieved BER of the system with LS is higher than the achieved BER of the system with ESE-OMP at the same SNR. This is because there is a determination of maximum correlation between the residual and sensing matrix columns in ESE-OMP. So, in ESE-OMP, LS is equipped with the determination of maximum correlation, and this process strengthens the decision of active device detection. It proves that LS cannot be used to estimate the number of active devices when these devices are communicating with BS without a scheduling process, such as in grant-free NOMA-IoT. In terms of complexity, ESE-OMP has a complexity of O(MNK), whereas LS has a complexity of O(MN). Therefore, ESE-OMP can be implemented in grant-free NOMA-IoT to estimate the number of active devices and detect the data of these active devices.

#### 6.1.2. Time Slot-Based Detection with Dynamic Composition of Active Device

**Figure 23:**
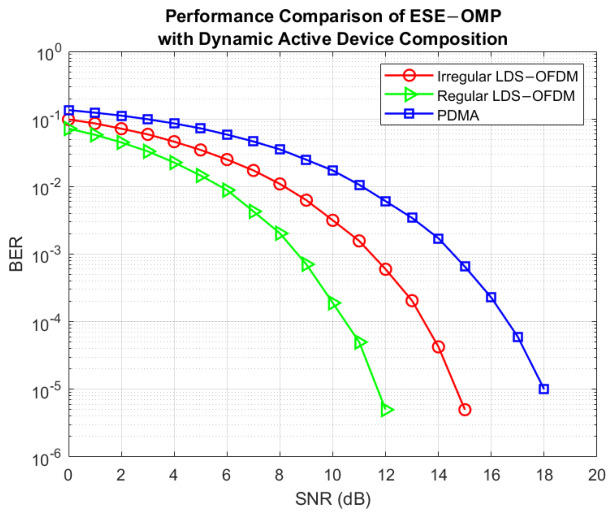
Performance comparison of ESE-OMP with dynamic active device composition for SMV problem with time slot-based detection. The systems evaluated are irregular LDS-OFDM, regular LDS-OFDM, and PDMA systems.

In this research, we also evaluate the performance of ESE-OMP with dynamic active device composition. In this scenario, the number of active devices, *K*, is 200. ESE-OMP can estimate the number of active devices correctly in all systems with an accuracy of 100%, as shown in Table 3. Figure 23 shows the performance of ESE-OMP in irregular LDS-OFDM, regular LDS-OFDM, and PDMA systems with dynamic active device composition. In this condition, device detection is carried out in a time slot, and the total number of active devices is constant. Sparse vector **s** is generated 1000 times with dynamic composition of active devices that are included in $C_{K}^{N}$ compositions. The performance of ESE-OMP in regular LDS-OFDM system is the best performance. To achieve a certain BER, regular LDS-OFDM with ESE-OMP needs a smaller SNR than irregular LDS-OFDM and PDMA systems with ESE-OMP.

#### 6.1.3. Time Slot-Based Detection with Dynamic Total Number of Active Devices

**Figure 24:**
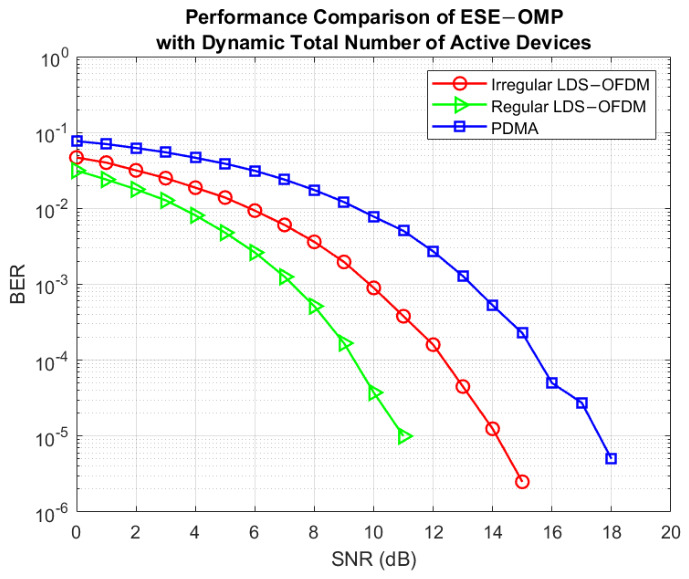
Performance comparison of ESE-OMP with dynamic total number of active devices for SMV problem with time slot-based detection. The systems evaluated are irregular LDS-OFDM, regular LDS-OFDM, and PDMA systems.

The SMV problem in this research also includes the dynamic total number of active devices. The performance of ESE-OMP in a system with 1000 sequential time slots and a varying number of active devices is evaluated. Variation of active device number is based on the value of *K* as shown in Table 1. The accuracy of sparsity level estimation for all systems is 100%, as shown in Table 4. ESE-OMP can estimate the number of active devices represented by the sparsity level correctly in regular LDS-OFDM, irregular LDS-OFDM, and PDMA systems. The performance of ESE-OMP with a dynamic total number of active devices is shown in Figure 24. The total number of active devices, *K*, varies, including 40, 80, 120, 160, and 200. In this condition, the composition of active devices also varies, and the performance of ESE-OMP in regular LDS-OFDM is also the best performance because of the subcarrier usage.

### 6.2. ESE-OMP Performance in MMV Problem

**Figure 25:**
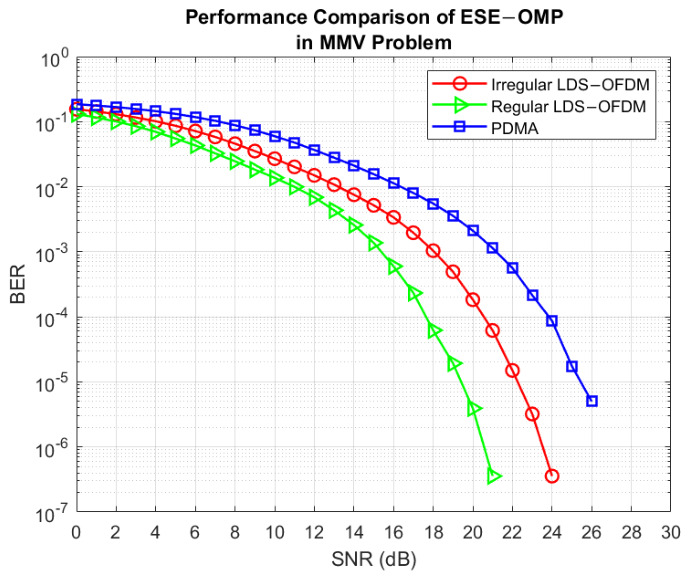
Performance comparison of ESE-OMP in frame-based detection as MMV problem. The systems evaluated are irregular LDS-OFDM, regular LDS-OFDM, and PDMA systems.

Communication with active device detection in a frame can be considered an MMV problem. In this case, the sparsity level that represents the number of active devices can be estimated correctly as shown in Table 5. The accuracy of sparsity level estimation is 100% for each system. The performance of ESE-OMP in regular LDS-OFDM system is the best performance, as shown in Figure 25. In regular LDS-OFDM system, each active device utilizes two subcarriers to communicate with the BS. Therefore, there are two subcarriers containing active device information. Once one subcarrier is decayed by the communication channel and AWGN, another subcarrier still contains the same information. Therefore, there is backup information that can be detected. In the MMV problem, all systems require a greater SNR than all systems in the SMV problem to achieve a certain BER. This is because there are 7 time slots that are detected in one time as a frame with the value of initial residual in the form of the average of the absolute value of measurement components, as shown in Figure 16. Once the number of time slots increases, the required SNR also increases to achieve a certain BER.

## 7. Conclusions

ESE-OMP can estimate the number of active devices and detect them correctly in SMV and MMV problems. ESE-OMP performance is the same as OMP performance in terms of SNR and BER, but ESE-OMP can estimate the number of active devices. In ESE-OMP, sparsity level estimation and signal reconstruction are carried out simultaneously. When ESE-OMP is implemented as device detection, the performance of the regular LDS-OFDM system is the best performance for all SMV and MMV problems, and the performance of the PDMA system is the worst performance. It is because all active devices in the regular LDS-OFDM system spread their information to two subcarriers, whereas each active device in the PDMA system spreads its information to a varied number of subcarriers. The performance of ESE-OMP for the SMV problem is better than the performance of ESE-OMP for the MMV problem. In static active device composition, the smaller the number of active devices, the better the performance of ESE-OMP. When the number of active devices increases, the SNR required by the system also increases to achieve a certain BER. Compared to LS, ESE-OMP can estimate the number of active devices communicating with BS correctly, whereas LS cannot estimate it. Therefore, ESE-OMP can be implemented as device detection in grant-free NOMA-IoT. For further research, the device detection method can be implemented in underwater or satellite communication with more active device numbers.

## Figures and Tables

**Table 1:** Simulation parameters.

Parameter	Value
*N*	400
*M*	300
*K*	40, 80, 120, 160, 200
*G*	7
Modulation type	BPSK
Frequency	2.3 GHz
Service type	Data

**Table 2:** Sparsity level estimation accuracy for static active device composition.

Number of Active Devices	System	Estimation Accuracy
40	Irregular LDS-OFDM	100%
	Regular LDS-OFDM	100%
	PDMA	100%
80	Irregular LDS-OFDM	100%
	Regular LDS-OFDM	100%
	PDMA	100%
120	Irregular LDS-OFDM	100%
	Regular LDS-OFDM	100%
	PDMA	100%
160	Irregular LDS-OFDM	100%
	Regular LDS-OFDM	100%
	PDMA	100%
200	Irregular LDS-OFDM	100%
	Regular LDS-OFDM	100%
	PDMA	100%

**Table 3:** Sparsity level estimation accuracy for dynamic active device composition.

System	Estimation Accuracy
Irregular LDS-OFDM	100%
Regular LDS-OFDM	100%
PDMA	100%

**Table 4:** Sparsity level estimation accuracy for dynamic total number of active devices.

System	Estimation Accuracy
Irregular LDS-OFDM	100%
Regular LDS-OFDM	100%
PDMA	100%

**Table 5:** Sparsity level estimation accuracy for MMV problem.

System	Estimation Accuracy
Irregular LDS-OFDM	100%
Regular LDS-OFDM	100%
PDMA	100%

## Data Availability

The original contributions are included in the article, further inquiries can be directed to the corresponding author.

## Abbreviations

The following abbreviations are used in this manuscript:

NOMA	non-orthogonal multiple access
IoT	internet of things
LDS-OFDM	low density spreading-orthogonal frequency division multiplexing
PDMA	pattern division multiple access
CS	compressive sensing
OMP	orthogonal matching pursuit
ESE-OMP	extended sparsity estimation-orthogonal matching pursuit
SMV	single measurement vector
MMV	multiple measurement vector
AWGN	additive white Gaussian noise
SNR	signal to noise ratio
BER	bit error rate
eMBB	enhanced mobile broadband
NR	new radio
mMTC	massive machine type communications
URLLC	ultra reliable and low latency communications
OMA	orthogonal multiple access
PD-NOMA	power-domain non-orthogonal multiple access
BS	base station
SCMA	sparse code multiple access
MP	matching pursuit
RIP	restricted isometry property
